# Prevention of Anti-microbial Peptide LL-37-Induced Apoptosis and ATP Release in the Urinary Bladder by a Modified Glycosaminoglycan

**DOI:** 10.1371/journal.pone.0077854

**Published:** 2013-10-30

**Authors:** Won Yong Lee, Justin R. Savage, Jianxing Zhang, Wanjian Jia, Siam Oottamasathien, Glenn D. Prestwich

**Affiliations:** 1 GlycoMira Therapeutics, Inc. Salt Lake City, Utah, United States of America; 2 Department of Medicinal Chemistry and Center for Therapeutic Biomaterials, University of Utah, Salt Lake City, Utah, United States of America; 3 Department of Surgery and Division of Pediatric Urology, University of Utah, Salt Lake City, Utah, United States of America; Northwestern University, United States of America

## Abstract

Interstitial cystitis (IC), often referred to in combination with painful bladder syndrome, is a chronic inflammatory disease of the bladder. Current therapies primarily focus on replenishing urothelial glycosaminoglycan (GAG) layer using GAG analogs and managing pain with supportive therapies. However, the elusive etiology of IC and the lack of animal models to study the disease have been major hurdles developing more effective therapeutics. Previously, we showed an increased urinary concentration of antimicrobial peptide LL-37 in spina bifida patients and used LL-37 to develop a mouse model of cystitis that mimics important clinical findings of IC. Here we investigate (1) the molecular mechanism of LL-37 induced cystitis in cultured human urothelial cells and in mice, (2) the protective effects of GM-0111, a modified GAG, within the context of this mechanism, (3) the physiological and molecular markers that correlate with the severity of the inflammation, and (4) the protective effects of several GAGs using these biomarkers in our LL-37 induced cystitis model. We find that LL-37 quickly induces release of ATP and apoptosis in the urothelium. These changes can be inhibited by a chemically-modified GAG, GM-0111. Furthermore, we also find that GAG analogs provide varying degrees of protection against LL-37 challenge in mice. These findings suggest that GM-0111 and possibly GAG molecules prevent the development of cystitis by blocking the apoptosis and the concurrent release of ATP from the urothelium.

## Introduction

Interstitial cystitis (IC) or painful bladder syndrome is a chronic disease characterized by clinical signs of bladder pain, frequent urination, and in some cases with Hunner’s ulcers [1]–[3]. The disease is fairly common with current estimates suggesting about 3 to 8 million US women ages 18 years or older suffer from the disorder [2]. IC may arise from multiple causes such as abnormal glycosaminoglycan (GAG) layer deficiency, urinary tract infection, neurogenic abnormality, immunological cause, leaky intercellular adhesion molecules, and possibly combination of multiple causes have been suggested [4]–[12]. The lack of mechanistic understanding of the disease has led to significant difficulties in diagnosing and treating IC, as well as for developing model systems to investigate the pathophysiology in order to develop more effective therapeutics [13].

In our previous studies, we found that intravesically instilling an antimicrobial peptide LL-37 at high concentrations could induce inflammation in the urinary bladder [14], [15]. The inflammatory phenotype underlying LL-37 induced cystitis exhibited various features observed in IC such as ulcerative lesions, edema, and the infiltration of leukocytes including mast cells in the bladder. Although LL-37 functions as part of the innate immune system, this peptide plays key roles in inflammatory signaling [16], [17]. Studies indicate that LL-37 exerts diverse biological effects by inducing apoptosis and attracting leukocytes [18]–[20]. The mechanism by which LL-37 induces inflammation in the bladder, however, remains unknown.

Current treatments for IC are very limited and mainly target supplementing the urothelial GAG layer using GAG molecules such as heparin and pentosan polysulfate [21]–[23]. One underlying hypothesis for using GAG molecules to treat cystitis is that these drugs can repair the defective GAG layer forming a barrier to cytotoxic urinary contents [4], [24]. Indeed, we showed that pre-treating the bladder with a modified GAG GM-0111 could prevent LL-37 induced cystitis [14]. By contrast, pre-treating the bladder with heparin provided negligible effects in reducing LL-37 induced cystitis. We speculate that the structural and biochemical differences between GM-0111 and heparin may be keys in preventing LL-37 induced cystitis.

In the present study, we investigate urothelial apoptosis and cellular ATP release as possible mechanisms of LL-37 induced cystitis, and we test the hypothesis that a modified GAG, GM-0111 can block both of these events and reduce the severity of cystitis. We also delineate physiological and biochemical measures that correlate with the severity of cystitis induced with LL-37. Using these objective measures, we demonstrate the protective effects of GM-0111 and compare the efficacy against other GAG analogs commonly used for IC treatment.

## Materials and Methods

### Study Compounds

GM-0111 was prepared as previously described [25]. Unfractionated heparin and chondroitin sulfate were purchased from Sigma-Aldrich (St. Louise, MO). Sodium pentosan polysulfate (Elmiron®) was obtained from IVAX Pharmaceuticals, Inc (Miami, FL). LL-37 is a C-terminal peptide fragment from human cathelicidin with a sequence of LLGDFFRKSKEKIGKEFKRIVQRIKDFLRNLVPRTES by single letter amino acid designation. The peptide was synthesized by the University of Utah HSC Core Research DNA/Peptide Facility and the purity was at or above 95%. All chemicals were dissolved in phosphate buffered saline (PBS, pH 7.4) and sterile filtered at 0.2 µm. Due to high viscosity, chondroitin sulfate was dissolved in sterile PBS and used without filtration.

### Animals

Female C57BL/6NCrl (C57BL/6) mice aged from 6–7 weeks were obtained from Charles River Laboratories (Wilmington, MA) and housed in an environment-controlled room at the University of Utah. The general health of the animal was monitored during an acclimatization period (at least one week). The feed and water were freely accessible during the entire period of time of the studies. The age of the animals at the time of treatments ranged from 8–11 wks.

### Ethics Statement

The studies were approved by the University of Utah Institutional Animal Care and Use Committee (Protocol number 11-08014) and all procedures were conducted according to the *Guide for the Care and Use of Laboratory Animals* [26].

### Intravesical Instillation of Drugs and LL-37

Study compounds and LL-37 were instilled in the urinary bladder similar to our previously described method [14]. Mice were anesthetized with isoflurane and a sterile silicone tubing (0.3 mm *i.d*.×0.64 mm *o.d.*, Dow Corning) lubricated with sterile glycerin was inserted into the urinary bladder. The bladder was then rinsed with PBS. The mice were then instilled with 150 µL of GM-0111, heparin, chondroitin sulfate, or pentosan polysulfate. To increase the contact of the bladder surface with the instilled compounds, we drained the compounds and re-instilled the same volume of each compound within 15 min after the initial instillation. After 1 hr of exposure to these compounds, the bladders were drained and equal volume of LL-37 (250 µM) was instilled for 1 hr to induce inflammation. To increase the contact of study compounds with the mucosal surface of the bladder, we also re-instilled LL-37 as in the compounds instillations. All instillation solutions were delivered at the speed of approximately 2 µL/sec to prevent vesicoureteral reflux. The catheter was then gently removed and the mice were allowed to recover. In a separate set of studies, the animals were challenged with 320 µM LL-37 for 1 hr and sacrificed at 1 hr and 3 hrs intervals to determine apoptosis in the urothelia.

### Necropsy and Microscopic Observations

To determine the protective effects of GM-0111 and other study compounds, the mice treated with these compounds were sacrificed 24 hr after the end of LL-37 instillation. First, the animals were anesthetized with isoflurane and the blood samples were collected. Next, the animals were euthanized by exsanguination through caudal vena cava and the urinary bladders were harvested after. The gross abnormality of the bladder was then scored according to the presence of hyperemia (0: normal and 1: hyperemic) and edema (0: none, 1–3 mild to marked). The sum of these scores served as the *necropsy score*. The bladders were then halved by transverse section and fixed in 10% formalin (Ted Pella, CA) for microscopic examinations. The remaining half of the tissue was stored at −20°C for biochemical analyses. Subsequent tissue processing for histological examination was carried out by Charles River Laboratory Histology Services (Wilmington, MA).

The severity of inflammation was scored in histological preparations stained with H&E similar to the previously described method by Johnson *et al* [25]. Each specimen was checked for the thickness of the urothelial layer (0, normal; 1, thin; 2, presence of denuded area), the extent of edema (0, none; 1, limited to submucosal region; 2, limited to submucosal region and the edematous area about twice thick as detrusor; 3, extended to near detrusor and the edematous area about 2–4 times the width of urothelium and detrusor combined; 4, even in the detrusor), and the infiltration of polymorphonuclear leukocytes (PMNs, 0, none to negligible; 1–3 from scant to marked). The sum of each measure served as the *histology score*.

### *In vitro* Studies

To determine the mechanism of LL-37 induced cystitis, human primary urothelial cells (HUCs, ScienCell Research Laboratories, CA) were plated in 24-well plates at a cell density of 5×10^5^/mL and grown overnight. The cells were then incubated with LL-37 to make final concentrations of 0, 0.3, 1.0, 3.0, 10, and 25 µM for 15 min at 37°C. In separate plates, overnight cultured cells were pre-incubated with GM-0111 at various concentrations for 30 min prior to LL-37 challenge (3 µM). The cell culture media was then collected and centrifuged at 12000 rpm for 5 min at 4°C to remove cells and cellular debris. The resulting supernatant was then used to measure the concentration of ATP using a luminescence based ATP determination kit (Life Technologies, NY) with a GloMax-Multi Microplate Multimode Reader (Promega, WI). In addition, cells were grown in 96-well plates at a cell density of 5×10^5^/mL overnight. These cells were then incubated with LL-37 as described above and the cellular caspase-3 and caspase-7 were directly measured from the plate using a luminescence-based Gaspase-Glo®3/7 assay kit (Promega, WI).

To measure apoptotic cell death by LL-37, we cultured HUCs in 24-well plates overnight at a cell density of ∼2×10^5^/mL. These cells were then incubated with GM-0111 for 30 min followed by 25 µM LL-37 challenge immediately after removing the cell culture media containing GM-0111. These cells were then stained for annexin V-FITC and 7-aminoactinomycin (7-AAD) for microscopy or trypsinized for flow cytometry analysis using Annexin V Apoptosis Detection kit (BioLegend, CA). Flow cytometry determination of Annexin V-FITC and 7-AAD positive cells was carried out using a Guava EasyCyte 8HT Flow Cytometer (EMD Millipore, MA). Data were analyzed with Flowing Software (version 2.5).

### Immunohistochemistry and TUNEL Staining

To visualize the extent of PMNs infiltration in LL-37 instilled bladders, we used rat monoclonal anti-Ly6G (or Gr-1) antibody (R&D Systems, MN) raised against native purified peptide from normal murine bone marrow cells. Ly6G is primarily expressed on the cell surface in the PMNs [26]. The IL-6 in the tissue was visualized with rabbit polyclonal anti-IL-6 antibody (abcam®, MA) raised against recombinant human IL-6 (homologous region with mouse IL-6). The presence of PTX-3 was visualized by using rabbit polyclonal anti-PTX-3 antibody (LifeSpan BioSciences, Inc., WA) raised against recombinant human PTX-3. Activated caspase-3 was tested by using rabbit polyclonal anti-caspase 3 antibody (LifeSpan BioSciences) that recognize the activated caspase-3 (p20 subunit). To retrieve antigens from the fixed tissues, we treated paraffin sections with either proteinase K (Ly6G) or with heat in citrate buffer (IL-6, PTX-3, activated caspase-3). The immunolabeled antigens were further labeled with ImmPRESS anti-rat Ig (Ly6G) and ImmPRESS anti-rabbit IgG (IL-6 and PTX-3) polymer detection kits (Vector Laboratories, CA) and visualized using ImmPACT DAB (Ly6G, PTX-3, and activated caspase-3) and ImmPACT VIP (IL-6) peroxidase substrates (Vector Laboratories, CA). Counterstain for nuclei was performed using Nuclear Fast Red (IHC WORLD LLC., MD) in PTX-3 immunolabeled tissues.

To investigate whether LL-37 induces apoptotic cell death in the urinary bladder, we visualized fragmented DNAs using Trevigen® TUNEL (terminal deoxynucleotidyl transferase dUTP nick end labeling) stain kit (Trevigen Inc., MD) and followed the manufacturer’s recommended protocol for staining.

### Biochemical Analysis of Tissue Homogenates

To determine the inflammatory response to LL-37 in the urinary bladder, we measured the tissue concentrations of various molecular markers. The frozen bladder tissues were first thawed on ice and immersed in ice cold lysis buffer (pH 7.4) that contained 200 mM NaCl, 10 mM Tris, 10% glycerin, 5 mM EDTA, and was supplemented with Halt protease inhibitor cocktail (Thermo Fisher Scientific, IL). The tissues were then homogenized by using zirconia/silica beads (BioSpec Products Inc., OK) at 4°C and cooled on ice for about 10 min. These tissue homogenates were subsequently centrifuged and the supernatants were collected for further analyses. ELISA assays were carried out to determine bladder tissue concentrations of IL-6 (BioLegend, CA) and PTX-3 (R&D Systems, MN). Blood levels of CRP and SAP were also determined via ELISA (Immunology Consultants Laboratory Inc., OR). The tissue activities of myeloperoxidase were determined by using the Fluoro MPO™ kit (Cell Technology Inc., CA). We also measured the concentration of total protein in each tissue homogenate supernatant using Bradford method using the Coomassie Plus Protein Assay (Thermo Fisher Scientific, IL) to normalize the tissue levels of molecules that we analyzed.

### Data Analysis

Measured body weight data from normal and LL-37 treated groups were calculated as a percent of the initial body weight (24 hrs prior to LL-37 treatment or equivalent time for normal animals). The individual weight of the urinary bladder was normalized to body weight. Tissue concentrations or activities of IL-6 and PTX-3, and MPO activity were adjusted to the concentration of total protein in the assay homogenates.

To determine whether pre-treating the urinary bladders with various concentrations of GM-0111, heparin, chondroitin sulfate, and pentosan polysulfate reduce the effects of LL-37, we analyzed the data using various statistical methods. The data of body weight changes, bladder weights, tissue concentrations and activities of IL-6, PTX-3 and MPO, and the serum concentrations of SAP were first tested for homogeneity of data using the Fligner-Killeen test [27] and the distribution of data were also visually inspected for parametric data analysis. The mean differences among various groups were determined with *one-way* analysis of variance test followed by Dunnett’s *t*-test by setting the data from normal animals or LL-37/PBS treated animals as control group. The data points fell under below detection limit (BDL) were substituted with calculated numbers: detection limit divided by , an imputation method used by Hornung and Reed [28]. The data of necropsy and histology scores along with data showing non-Gaussian distribution were analyzed with Kruskal-Wallis rank sum test followed by a multiple comparison test. The *post hoc* multiple comparison test for Kruskal-Wallis rank sum test was done by using the ‘*pgirmess’* package written by Patrick Giraudoux for R statistical software [29]. Spearman’s rank correlation was measured to investigate correlations between physiological measurements and biochemical markers and the resulting coefficient (Spearman’s ρ) was calculated. The statistical significance was set to *p*<0.05. All data were analyzed using R statistical software version 2.15.

## Results

### LL-37 Induces Apoptosis in the Bladder

**Figure 1:**
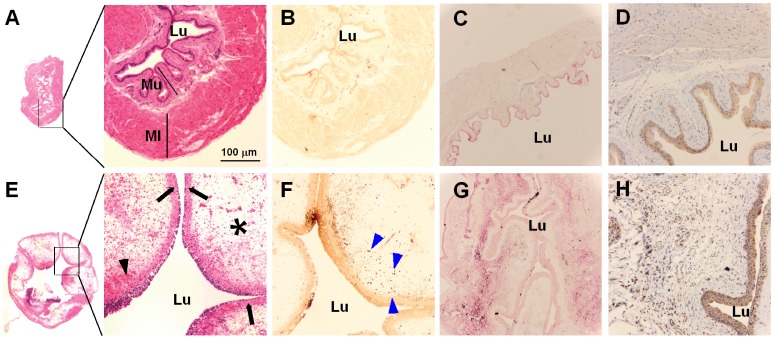
LL-37 induces inflammatory changes in the urinary bladder (A, B, C, and D: normal bladder; E, F, G, and H: 24 hr after 250 µM LL-37 treatment). Microscopic observations show that LL-37 causes edema (*), massive infiltration of leukocytes including PMNs and occasional hemorrhage (black arrow head) along with urothelial erosion (arrow). A and E are photostiched images of the entire bladder surface stained with H&E (lower panels are the enlarged views of the square regions). B and F are immunostained images for Ly6G, the surface antigen from PMNs (blue arrowheads). C and G are immunostained images for IL-6 showing strong immunoreactivities of IL-6 at the submucosal area in the LL-37 treated bladder. D and H are immunostained images for PTX-3 showing marked increase of PTX-3 positive cells in the LL-37 treated bladder. Lu: lumen, Mu: mucosa, ML: muscle layer.

**Figure 2:**
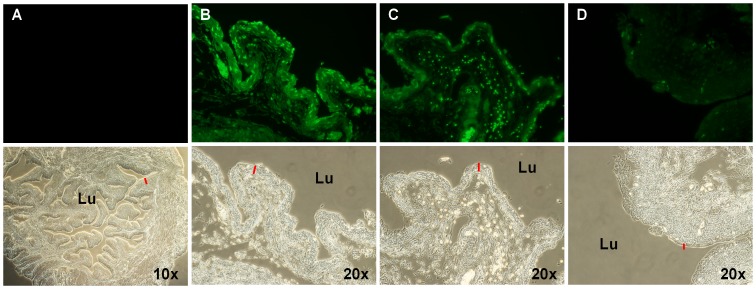
LL-37 induces apoptotic cell death in the urinary bladder. The mucosal cells in the urinary bladder undergo apoptotic cell death and are removed quickly. TUNEL stain (top panels) and corresponding bright field (bottom panels) images of bladders from (A) normal animal, (B) normal bladder treated with TACS nuclease for positive control, (C) 1 hr after LL-37 (320 µM) challenge, and (D) 3 hr after LL-37 challenge. Red lines indicate the urothelial layers. Lu is the lumen of the bladder.

We have previously shown that intravesically instilled LL-37 induces acute inflammation in the urinary bladder similar to Hunner’s lesions found in interstitial cystitis (IC) patients [1], [14]. LL-37 induced cystitis is characterized by marked edema in all layers of the bladder (Fig. 1A *vs*. 1E), infiltration of polymorphonuclear leukocytes (PMNs, Fig. 1B *vs*. 1F), and ulcerative lesions in the mucosa (Fig. 1A *vs*. 1E). While the cause of this chronic inflammatory changes in IC bladders remain elusive, studies have shown an increased number of apoptotic cells from IC patients [9], [30]–[32]. LL-37 has been known to induce apoptosis in various cell types, suggesting that LL-37 may cause the inflammatory signaling by inducing apoptosis in the bladder [17], [19], [20], [33]–[36]. To test whether LL-37 induces apoptosis in the bladder, we intravesically instilled LL-37 into the mouse bladders, harvested them 1 hr and 3 hrs later, and processed the tissues to visualize fragmented DNAs with terminal deoxynucleotidyl transferase dUTP nick end labeling (TUNEL) stain. The harvested tissues from 1 hr after LL-37 challenge showed an increased number of TUNEL positive cells in the mucosal and submucosal layers compared to the bladders harvested from normal mice (Fig. 2). These changes quickly subsided as there were fewer TUNEL positive cells in bladders harvested 3 hr after LL-37 challenge (Fig. 2).

**Figure 3:**
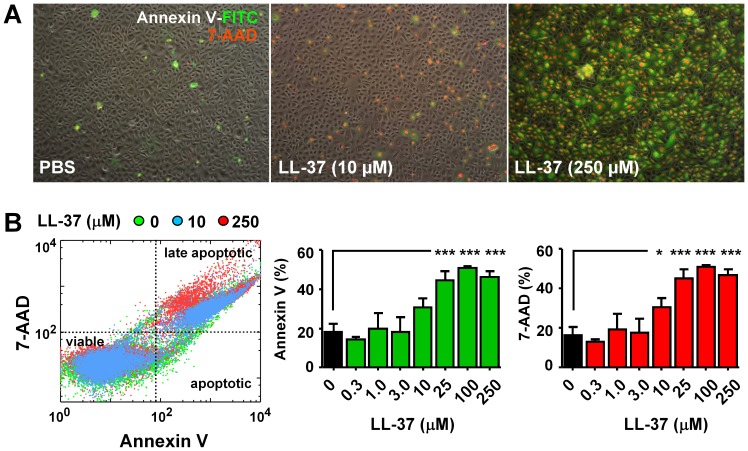
LL-37 induces apoptotic cell death in human urothelial cells (HUCs). Cultured HUCs were treated with LL-37 for 15 min at 37°C. HUCs were then collected and labeled with annexin V-FITC (shown in green) and 7-AAD (shown in red). A, Fluorescence microscopy shows the increased number of apoptotic cells in LL-37 treated HUCs (magnification 10*x*). B, Flow cytometry analysis of HUCs shows that LL-37 induces apoptotic cell death at ≥ 10 µM or higher concentrations of LL-37. *p<0.05 and ***p<0.001 by Dunnet’s *t*-test.

To verify that LL-37 induces apoptosis in the bladder, we measured annexin V binding on the cell surface and 7-AAD fluorescent dye uptake in the cultured human primary urothelial cells (HUCs) stimulated with LL-37 at various concentrations. HUCs exposed to LL-37 showed increased uptake of 7-AAD that are also annexin V positive (Fig. 3A). Flow cytometry analysis confirmed that LL-37 dose-dependently increased apoptotic cells with the response reaching maximum at around 25 µM of LL-37 (Fig. 3B).

Multiple mechanisms appear to be involved in LL-37 induced apoptosis that are either caspase-dependent or caspase-independent varying by the type of the tissues [19], [20], [33]. To determine whether LL-37 induces apoptosis in a caspase-dependent manner, we immunolabeled activated (cleaved) caspase-3 in the LL-37 challenged bladder tissue. We found no evidence for immunoreactivity against activated caspase-3 (data not shown). In addition, the activities of caspase-3 and caspase-7 in cultured HUCs challenged with various concentrations of LL-37 were unchanged (data not shown). These data suggest that LL-37 induces apoptosis in a caspase-independent mechanism within the urothelia.

### LL-37 Induces ATP Release from Urothelial Cells

**Figure 4:**
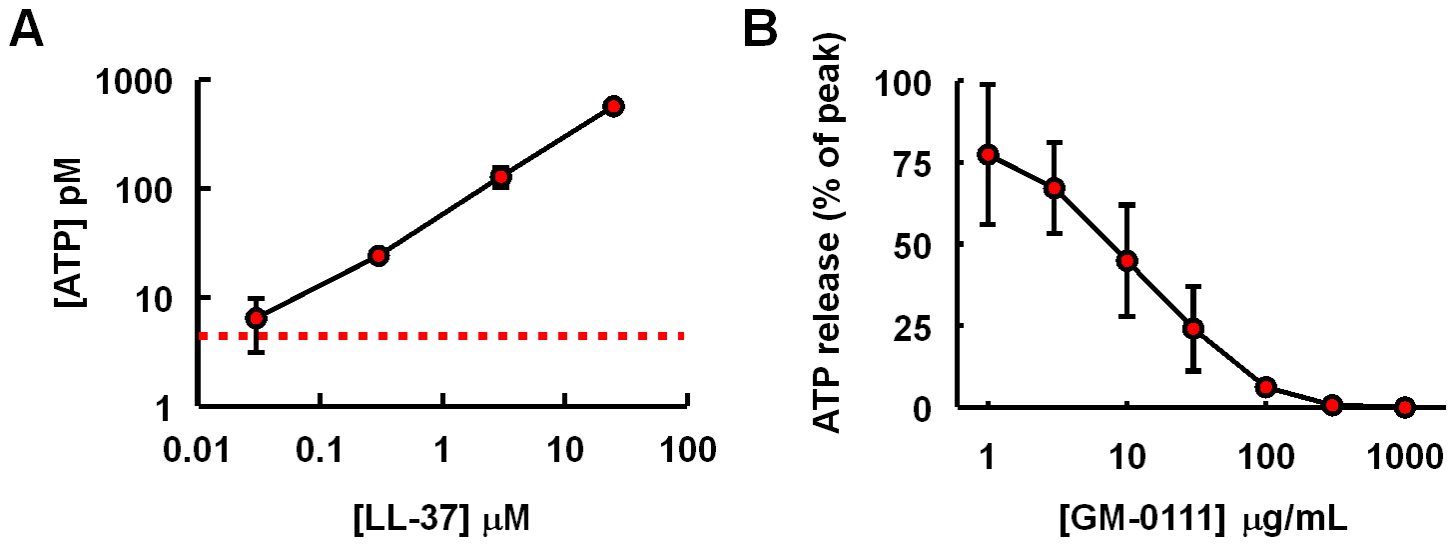
LL-37 induces ATP release from human urothelial cells (HUCs) (A) and GM-0111 reduces LL-37 induced ATP release in a dose-dependent manner (B). HUCs were challenged with LL-37 at various concentrations for 15 min and the culture supernatants were analyzed for ATP. To determine the inhibitory effects of GM-0111 against LL-37 induced ATP release, HUCs were treated with GM-0111 for 30 min prior to the 15 min LL-37 challenge (3 µM). Dotted red line indicates the basal concentration of ATP in the cell culture supernatant.

At sufficiently high concentration, LL-37 induces apoptotic cell death in the urothelial cells lining the bladder. How does this event relate to the inflammatory changes in the bladder? Urothelial cells undergoing apoptosis may release signaling molecules that activate early inflammatory mediators. One such molecule is adenosine triphosphate (ATP). Urothelial cells release ATP as a physiological response to the stretch of the bladder by increasing urine volume [37], [38]. Released ATP activates purinergic receptors such as P2X3 that contribute to pelvic afferent fibers [39]. ATP is also an important mediator of early inflammatory signaling by activating P2X7 receptors that function to process and release pro-inflammatory cytokines such as IL-1β [40]. We tested the possibility of LL-37 inducing ATP release in cultured HUCs. There was a marked increase in ATP release when HUCs were stimulated with LL-37 at 300 nM or higher (Fig. 4A). These data suggest that LL-37 may trigger early inflammatory signaling by releasing ATP from urothelial cells.

### GM-0111 Reduces LL-37 Induced Cystitis

**Figure 5:**
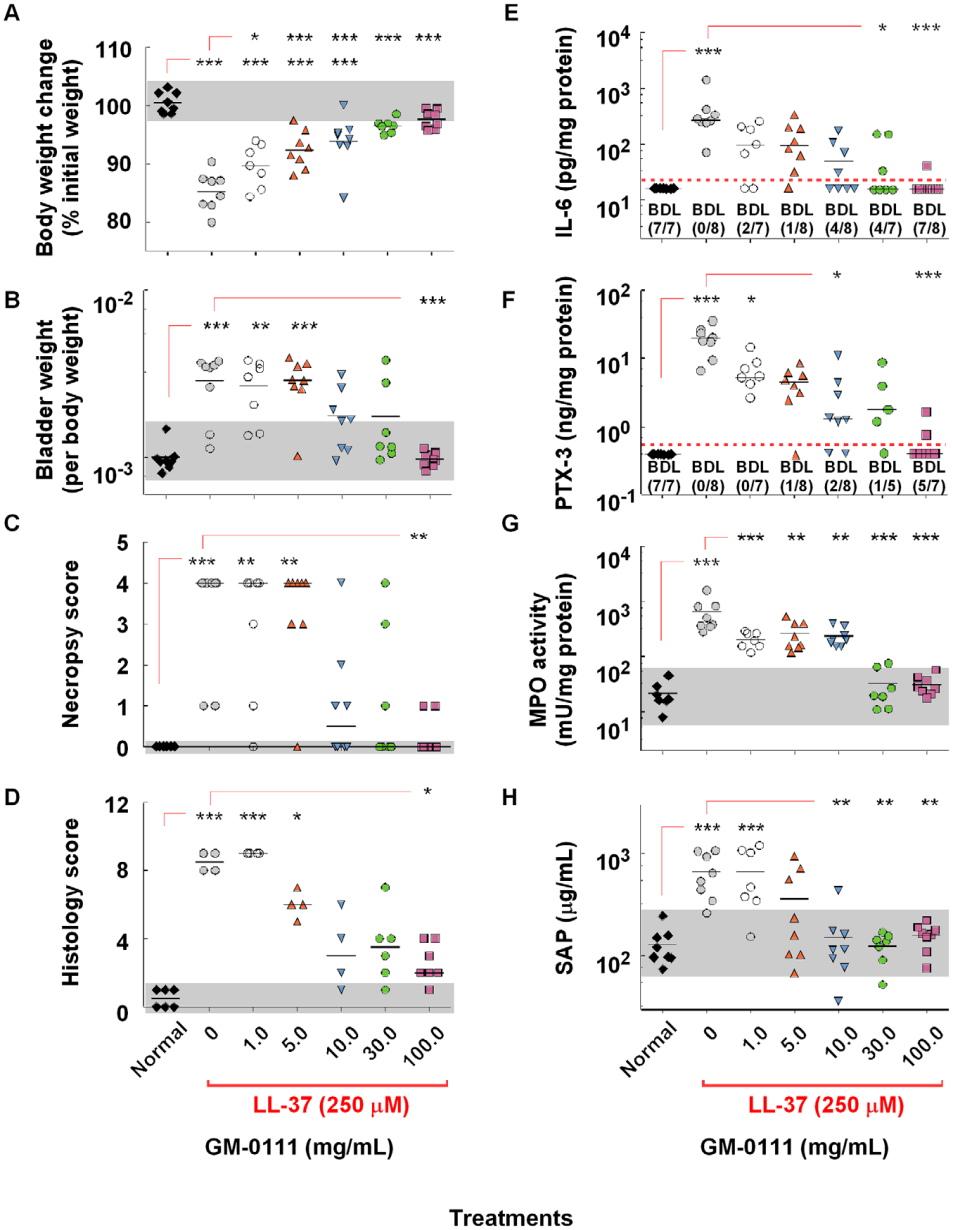
LL-37 instillation leads to local and systemic inflammatory changes and GM-0111 reduces the inflammatory effects of LL-37. The animals treated with LL-37 show decreased body weight gain (A), increased bladder weight (B), gross anatomical changes in the bladder (C), severity of inflammatory changes in histological observations (D), and the bladder concentration of IL-6 (E), PTX-3 (F), MPO activity (G) and blood level of SAP (H). *p<0.05, **p<0.01, and ***p<0.001 compared to Normal or PBS pre-treatment/LL-37 treated control. Black horizontal bars are mean or median (necropsy and histology scores) values. BDL and the values in the parenthesis represent the number of samples that fell below the detection limit (dotted red line) of the ELISA out of the total samples tested (see Data Analysis in Materials and Methods section for details). Gray area represents the range of values found in normal animals.

Previously, we reported that instilling a modified GAG, GM-0111 into the bladder could reduce the cystitis induced by instilling LL-37 into the bladder [14]. To delineate the impact of LL-37 induced cystitis not only in the bladder but also systemically, we investigated molecular and physiological markers that were altered by the developing cystitis. In addition, we also evaluated the effectiveness of GM-0111 in reducing the LL-37 induced cystitis by measuring these biomarkers. For physiological changes, we measured body weight changes and bladder weight at necropsy. Mice intravesically instilled with PBS prior to LL-37 challenge showed significant loss of body weight (∼15%) within 24 hr (Fig. 5A). The bladders were edematous (Fig. 1B) and markedly increased in size weighing approximately 3 times that of the non-LL-37 challenged controls (Fig. 5B). Pre-treating the bladder with GM-0111 reduced these physiological and morphological changes in a dose-dependent manner (Fig. 5A–5D). For molecular markers in the tissues, we considered proteins directly released from cells involved in the inflammatory responses. Histological observations indicated that LL-37 induces infiltration of various leukocytes with increased vascularization [14]. Both leukocytes and endothelial cells release various cytokines and proteins upon inflammatory signaling. Our pilot studies of biochemical analysis showed higher concentrations of various cytokines in the tissues such as IL-6. The major cellular sources of IL-6 are macrophages, granulocytes, and myocytes. IL-6 has been used to measure the inflammatory response in the tissues from other cystitis animal models and the increased levels of urinary IL-6 were also reported as a possible biomarker for IC [40]–[46]. Immunohistochemistry for IL-6 showed strong immunoreactivity of IL-6 in the submucosal layers (Fig. 1G) and the tissue concentration was highly dependent on the dosage of GM-0111 (Fig. 5E). We also investigated a possible change of PTX-3 in the tissue, which is a member of the pentraxin family and plays important roles in inflammation [47]–[49]. The cellular sources of PTX-3 are diverse but neutrophils and endothelial cells are the likely main source during early phases of inflammation [48], [50]–[52]. We speculated that this protein might serve as a dependable indicator of inflammation. Immunohistochemistry for PTX-3 showed strong immunoreactivities of PTX-3 in all tissue layers (Fig. 1H) of the LL-37 challenged bladders while the expression levels in normal bladders were minimal. The tissue concentrations of PTX-3 were also strikingly higher in the LL-37 challenged bladders than in normal controls (Fig. 5F). Similar to tissue IL-6, the PTX-3 level in the bladder was also inversely dependent on the dosage of GM-0111. Previously, we measured tissue concentrations of myeloperoxidase (MPO) to determine the level of cystitis. MPO is a neutrophilic granular protein and the tissue level of MPO has been widely used to quantitatively measure the degree of neutrophilic infiltration at the site of inflammation [53]. In our studies, we determined the enzymatic activity of MPO in the bladders. Consistent to our previous studies, the MPO activity in LL-37 challenged bladders was higher than in normal tissues (Fig. 5G) [14]. GM-0111 pre-treatment also reduced MPO activity in the bladders challenged with LL-37. These data suggest that both IL-6 and PTX-3 are highly reflective of the severity of the cystitis.

While molecular markers in the tissues such as IL-6, PTX-3, and MPO are useful in delineating the severity of inflammation at the organ, it would be highly convenient and useful to have a molecular indicator in the blood that correlates with the state of inflammation in the bladder. Both serum amyloid P (SAP) and C-reactive protein (CRP) are acute phase proteins synthesized by the liver and have been widely studied to monitor various inflammatory diseases [54]–[56]. We investigated whether these proteins could be used to monitor the severity of cystitis induced with LL-37. The mean blood levels of SAP increased 5-fold in the LL-37 challenged group compared to the normal animal group (Fig. 5H). The anti-inflammatory effects of GM-0111 also appeared to follow the results of the other molecular markers in the bladder tissues. By contrast, the blood levels of CRP were unchanged with LL-37 challenge (data not shown). These findings are consistent to previous reports that SAP but not CRP more likely increase upon inflammation in rodents [57]–[59]. These data suggest that SAP can be a molecular indicator in the blood to monitor the severity of LL-37 induced cystitis.

**Figure 6:**
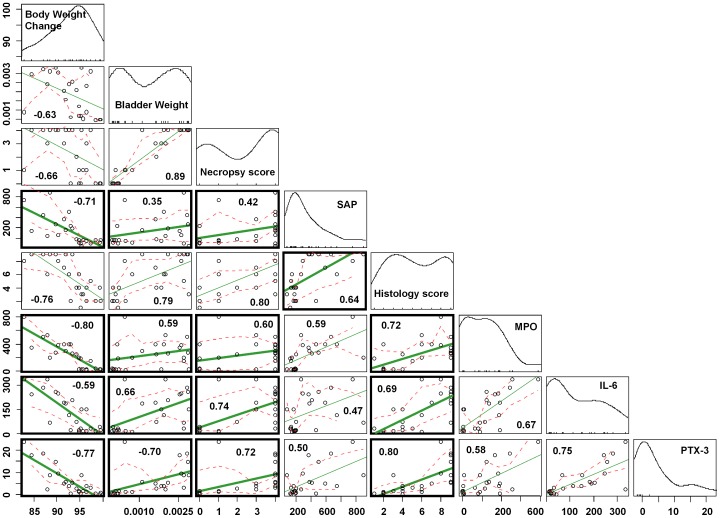
Multivariate analysis of various observations in LL-37 induced cystitis model and the inhibitory effects of GM-0111. Each intersecting graph shows the correlation between the two respective observational parameters. Graphs in the diagonal array represent the distribution of data from each parameter pooled from normal and LL-37/GM-0111 treated animals. Green lines indicate the correlation in each set of parameters and red dotted lines indicate the values following similar trends. Bold rectangular areas represent the correlation between physiological measurements and the concentrations of molecular markers. The values inside boxes are Spearman correlation coefficients (ρ).

The overall data suggest that the tissue and blood markers in our studies are consistent with the physiological and morphological measurements to determine the severity of LL-37 induced cystitis. Are there any correlations among these various measurements? Multivariate analysis showed that certain measurements such as body weight changes highly correlated with the blood level of SAP, tissue MPO activity, and PTX-3 concentrations (Fig. 6). Histology scores showed strong correlation with blood levels of SAP and tissue MPO, IL-6 and PTX-3. Bladder weight correlated well with bladder concentration of IL-6 and PTX-3. Overall, these data suggest that combined measurements of physiological and biochemical markers are useful to evaluate the severity of cystitis and to test the anti-inflammatory effects of potential therapies such as GM-0111.

### GM-0111 is Effective in Preventing Cystitis

**Figure 7:**
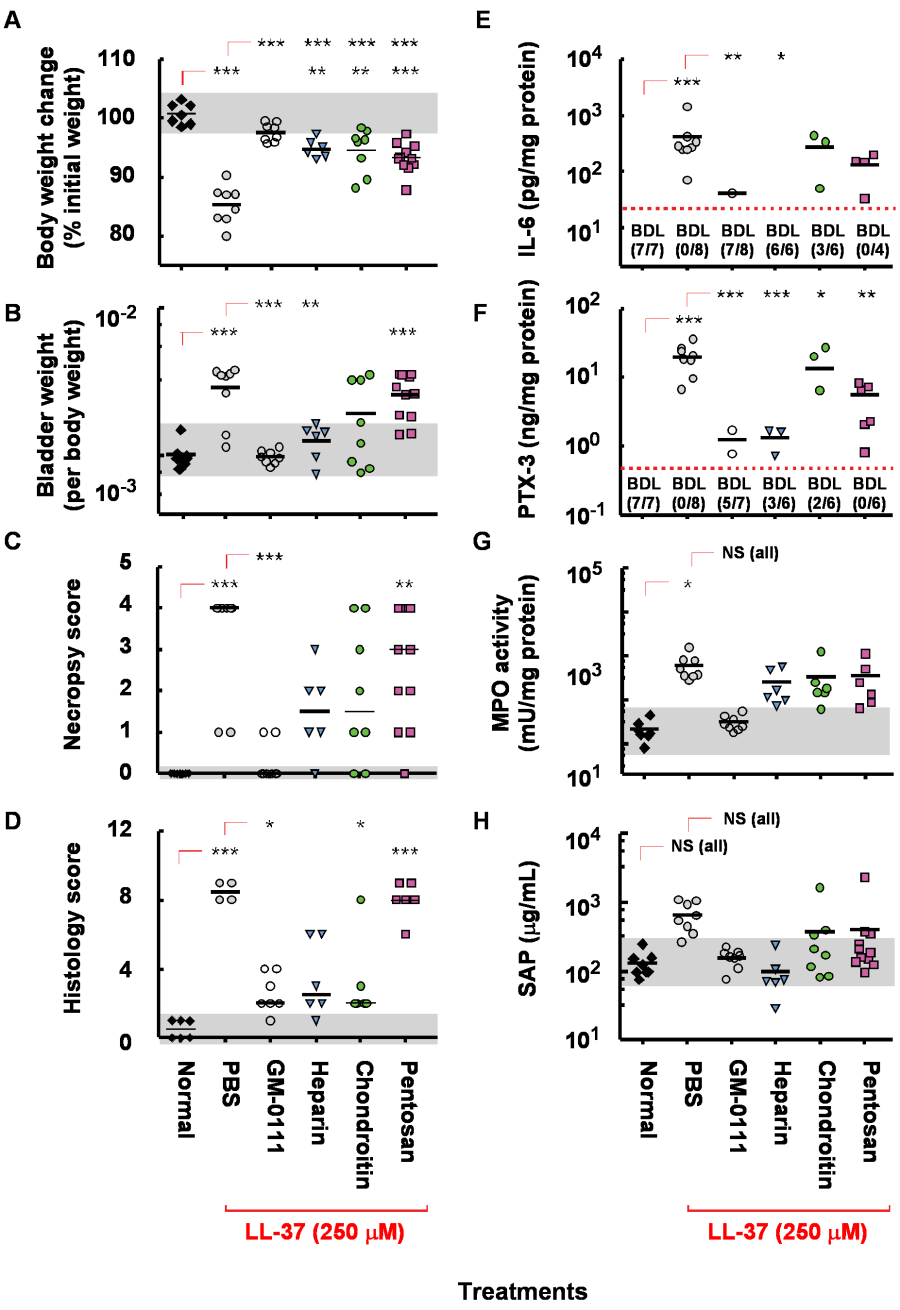
The protective effects of GM-0111 and heparin against LL-37 induced cystitis are more powerful than chondroitin sulfate or pentosan polysulfate. The animals receiving each compound at 100/mL dosage by intravesical instillation prior to LL-37 instillation showed markedly reduced clinical signs of IC and decreased molecular markers in the serum and tissues. *p<0.05, **p<0.01, and ***p<0.001 compared to either normal or PBS/LL-37 instilled control group. NS, not significant (*p*>0.05) by Dunnet’s t-test. Black horizontal bars are the mean or median (necropsy and histology scores) values. BDL and the values in the parenthesis represent the number of samples that fell below the detection limit (red dotted line) of the ELISA out of the total samples tested. For data analysis purposes, any values below the detection limit were listed as being at the detection limit, which may in some cases overestimate the numerical value. Gray area represents the range of values found in normal animals.

**Figure 8:**
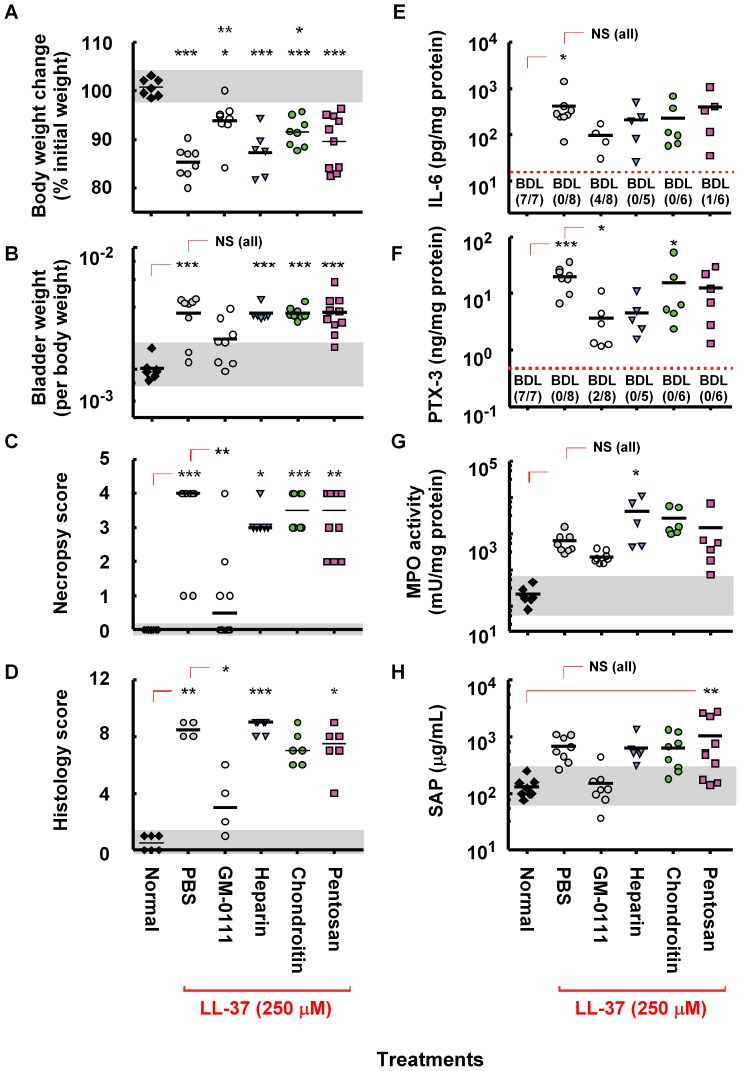
The protective effects of GM-0111 against LL-37 induced cystitis are more powerful than other GAG compounds. The animals receiving each compound at 10/mL dosage by intravesical instillation prior to LL-37 instillation showed variable responses in clinical signs of IC and decreased molecular markers in the serum and tissues. Only the animals treated with GM-0111 were effectively protected from the LL-37 challenge. *p<0.05, **p<0.01, and ***p<0.001 compared to either normal or PBS/LL-37 instilled control group. NS, not significant (*p*>0.05) by Dunnet’s *t*-test. Black horizontal bars are mean or median (necropsy and histology scores) values. BDL and the values in the parenthesis represent the number of samples that fell below the detection limit (red dotted line) of the ELISA out of the total samples tested. For data analysis purposes, any values below the detection limit were listed as being at the detection limit, which may in some cases overestimate the numerical value. Gray area represents the range of values found in normal animals.

Our collective data suggest that GM-0111 is effective in reducing the inflammatory changes induced by LL-37. Various GAG analogs such as heparin, pentosan polysulfate and chondroitin sulfate (Table S1) are currently available to treat IC. Our previous studies showed that GM-0111 is more effective in reducing LL-37 induced cystitis at 10 mg/mL [14]. How does GM-0111 fare in protecting LL-37 induced cystitis compare to other GAG compounds that are currently used for IC? To evaluate the efficacy of GM-0111, we intravesically instilled heparin, chondroitin sulfate, or pentosan polysulfate at 2 different dosage levels, challenged the pretreated bladders with LL-37, and evaluated the changes of the physiological and biochemical markers. At a dosage of 100 mg/mL, the protective effects of heparin and chondroitin sulfate were similar to GM-0111 while PPS showed poor protection against LL-37 induced cystitis (Fig. 7). By contrast, the animals pre-instilled with 10 mg/mL of each compound showed marked differences in effectiveness in preventing LL-37 induced cystitis (Fig. 8). GM-0111 demonstrated the highest level of protection of the bladder against LL-37 induced cystitis in most physiological and biochemical measures. These data suggest that GM-0111 is more effective than other GAG compounds in reducing cystitis. In addition, the physiological and biochemical measures used in our studies showed clear differences in the efficacies of these compounds that were difficult to estimate previously.

### GM-0111 Reduces LL-37 Induced Apoptosis and ATP Release by Urothelial Cells

**Figure 9:**
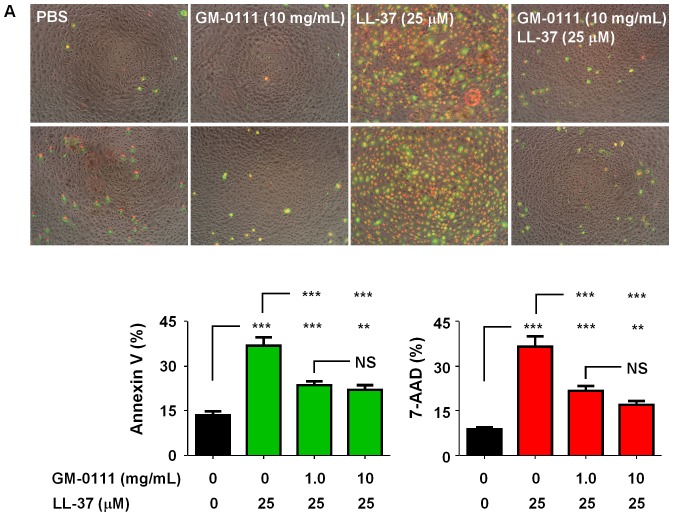
GM-0111 reduces LL-37 induced apoptosis in human urothelial cells (HUCs). HUCs pretreated with GM-0111 at 0, 0.1, 1.0, and 10 mg/mL for 30 min were challenged with LL-37 (25 µM) for 15 min at 37°C. Cells were stained with annexin V-FITC (shown in green) and 7-AAD (shown in red) to visualize (A) with microscopy (magnification 10*x*) and to quantify (B) the apoptotic cells with flow cytometry. **p<0.01, ***p<0.001 and NS, not significant (*p*>0.05) by Dunnet’s *t*-test.

How does GM-0111 reduce the cystitis induced by LL-37? Our data indicate that LL-37 induces ATP release and apoptosis in urothelial cells that likely contribute to the development of cystitis. To investigate whether GM-0111 can block these molecular events, we treated HUCs with GM-0111 followed by LL-37 challenge. And then we determined apoptosis and ATP release by HUCs. Both microscopy and flow cytometry analysis data showed that GM-0111 was able to reduce LL-37 induced apoptosis (Fig. 9). In addition, GM-0111 could block LL-37 induced ATP release from HUCs (Fig. 4B). These data suggest that GM-0111 may protect the bladder from LL-37 induced the inflammatory assault by reducing apoptosis and ATP release from the urothelia.

## Discussion

Due to the unknown cause of IC, developing drugs to treat the disease face major challenges. Important among these challenges is the absence of a consensus on the most physiologically relevant animal models, and the diagnostic and outcome criteria important for establishing the effectiveness of any new drugs [59]. Recent studies of genomic and proteomic approaches suggest that IC has molecular features of both inflammatory and neuronal signaling [60]. Increased tissue levels of inflammatory mediators, cytokines, and high blood levels of acute phase protein such as C-reactive protein were found in IC patients [47]. Neuronal signaling mediators that were increased in IC patients include nerve growth factor (NGF) and brain-derived neurotrophic factor (BDNF) [47]–[49]. In addition, increased urine or tissue levels of antiproliferative factor (APF), adenosine triphosphate (ATP), vascular endothelial growth factor (VEGF), and increased apoptotic cells were reported suggesting that the pathogenesis of IC involves abnormalities in the GAG layer, urothelial permeability, inflammatory signaling, and increased pain sensation [7], [9], [54], [55], [61].

LL-37 induced cystitis shares many of the inflammatory changes found in IC. Ulcerative lesions with marked edema and massive infiltration of leukocytes throughout all layers of the bladder caused by LL-37 concentrations at or above 250 µM are similar to the Hunner’s lesions found in a subset of IC [1]. Increased concentrations of LL-37 in the urine of pediatric spina bifida patients were recently reported [15]. Given that these patients frequently suffer from chronic bladder inflammation, LL-37 is likely physiologically relevant [56]. It is notable that increased LL-37 is found in the synovium of rheumatoid arthritis patients [16]. However, it is yet to be determined whether LL-37 is also increased in all or some subset of IC patients.

How does LL-37 induce inflammatory changes in the bladder? The rapid apoptosis of the urothelium may be a key to the early development of inflammation. Apoptotic and anti-apoptotic effects of LL-37 have been described in various tissues and the accumulating data suggest that the effects are dose-dependent. At high concentration (≥10 µM or ∼2.2 µg/mL in urothelial cells), LL-37 becomes apoptotic in urothelial cells as shown in our studies and in Jurkat cell studies [33]. In both cases, the apoptosis seems to take a caspase-independent pathway. The apoptosis and subsequent quick removal of dead urothelial cells is likely the reason that a thinner urothelial layer is observed in the histological preparations from LL-37 treated bladders. Recent studies have shown increased apoptotic urothelial cells in IC patients suggesting that apoptosis in the urothelium may be a part of the pathogenesis of IC [30], [32].

In parallel to the apoptosis, LL-37 activates or modulates various inflammatory signaling pathways. The known molecular targets of LL-37 are diverse. G-protein coupled receptors such as FPR2, IL-8RB, MrgX2, and P2Y11, and a ligand-gated ion channel P2X7 have been suggested as possible receptors for LL-37 in various eukaryotic cells such as leukocytes, neurons, and urothelial cells. These receptors mediate inflammatory responses by activating cytoplasmic targets to release cytokines, control cell motility, and cause cell proliferation [62], [63]. Studies suggest that LL-37 forms pores in the cell membrane that lead to ATP release from the cell [64], [65]. ATP is an important factor in bladder physiology as a normal bladder releases ATP in response to the bladder distension caused by urine. The released ATP activates P2X3 purinergic receptors present on the sensory organs to mediate activation of afferent nerves leading to the contraction of the bladder muscle [66]. It is conceivable that the high concentration of ATP occurring in the urine of IC patients may be a critical factor in causing frequent urination and pain, and studies to explore this possibility will be undertaken. Emerging evidence suggests that ATP plays key roles in the early development of inflammation by activating and releasing IL-1β through P2X7 receptors [23], [40], [43], [63], [67], [68]. The inhibitory effects of GM-0111 and possibly other GAGs on ATP release as shown in our study likely are a contributing mechanism to the prevention of the development of cystitis induced with LL-37.

Treatment options for IC patients that are currently approved by the US FDA or are under investigation such as Elmiron (pentosan polysulfate), Uracyst (chondroitin sulfate), and URG 101 (heparin and lidocaine) target the GAG layer and pain sensation. GM-0111 is a semi-synthetic GAG based on chemically-modified hyaluronic acid; its anti-inflammatory properties are equal or superior to those of heparin, but it is over 100-fold less anti-coagulant than heparin [69]. Our previous studies suggested that GM-0111 was more effective in preventing inflammation than heparin in mouse models of cystitis and rosacea [14], [70]. We also showed that GM-0111 could bind to the bladder wall and it even penetrated submucosal layers [14]. In our current study, we further investigated the inflammatory changes of the urinary bladder and the whole body induced with LL-37. Major challenges in evaluating the efficacy of a drug to treat IC are lack of biomarkers. Therefore, it is promising to see studies showing various molecular markers found in IC patients as noted above. Our studies showed that intravesical instillation of LL-37 caused significant weight loss within 24 hr. Body weight changes are important in determining the overall health of the animal [67]. We suspect the body weight losses observed in LL-37 treated animals are due to a combination of abdominal pain, lack of appetite, or reduced water intake by the animals reflecting the inflammation of the bladder. Molecules such as IL-6, PTX-3, and MPO present in the bladder tissues indicate the inflammatory responses from various cellular sources. We found that the tissue concentrations and the activities of these molecules highly correlated with the body weight changes suggesting that they could serve as good biomarkers in the LL-37 induced cystitis model. Serum amyloid P is an acute phase protein and has served as a good indicator of inflammatory changes in the body. The increased blood level of SAP in the LL-37 treated animals suggests that this molecule may be a valuable indicator to continuously monitor the disease progression in live animals.

One of the major aims of our study was to compare the effectiveness of GM-0111 in preventing cystitis with drugs that are used in the clinic namely heparin, pentosan polysulfate, and chondroitin sulfate. We did not include hyaluronic acid (HA) in our study because the efficacy of HA was poor compared to GM-0111 in various molecular targets (unpublished results). Dose-response studies showed that the early signs of effectiveness of GM-0111 appeared in dosages as low as 1 mg/mL and became more apparent at 10 mg/mL reaching maximum protection at 100 mg/mL in all physiological and biochemical measures. While comparable protective effects were observed in animals pretreated with heparin at 100 mg/mL dosage, the effects of heparin at 10 mg/mL were much less than the effects of GM-0111 at the same dosage level. In addition, the effects of chondroitin sulfate and pentosan polysulfate were very poor compared to heparin and GM-0111. Previous studies have shown that all of these drugs are able to reduce inflammatory changes in various cystitis models. These studies relied on clinical signs and histological observations to assess the effectiveness of the drugs [21], [23], [71]. Our studies also support that these drugs are effective in histological evaluations. However, the comprehensive measures with physiological and molecular assessment as in our studies suggest that the protective effects of heparin, chondroitin sulfate, and pentosan polysulfate are less than perfect when compared to GM-0111. These data suggest that GM-0111 will likely provide more powerful protection against inflammatory changes in the bladder.

How does GM-0111 inhibit LL-37 induced cystitis? Similarly to other GAG analogs, GM-0111 likely provides anti-inflammatory effects in the bladder by multiple mechanisms. One possibility is fortifying the protective barrier of the urothelium as a GAG analog. Previously, we showed that GM-0111 can coat and even penetrate the deeper layers of the bladder mucosa thereby supporting this mechanism [14]. Another possibility is that the polyanionic GAG GM-0111 may directly neutralize toxic molecules in the urine through electrostatic interactions with LL-37 and other cationic molecules. The degree of sulfation and the resulting negative charge in GM-0111 and pentosan polysulfate are similar but the protective effects of pentosan are much poorer than GM-0111 or less negatively charged heparin or chondroitin sulfate. These suggest that neutralizing cations in LL-37 are not the sole mechanism of GM-0111 to protect the bladder from LL-37 challenge. GM-0111 may further provide its anti-inflammatory effects by directly inhibiting inflammatory mediators such as selectins, danger-associated molecular pattern (DAMP) molecules, and leukocyte elastase as shown in previous studies [70]. In addition, GM-0111 inhibits the release of ATP and reduces apoptosis in urothelial cells suggesting that these effects are also contributing mechanisms to protect the bladder from developing cystitis.

## Conclusion

LL-37 induces apoptosis in the urothelia that mediates the inflammatory changes in the bladder. These changes can be monitored and quantified by measuring physiological and biochemical markers that are altered by the inflammatory stimuli. GM-0111, a semi-synthetic GAG provides a powerful protection to the bladder against LL-37 induced cystitis by inhibiting apoptosis and the release of ATP in the urothelium.

## Supporting Information

Table S1 **Chemical structures of glycosaminoglycan analogs.**
(DOCX)
