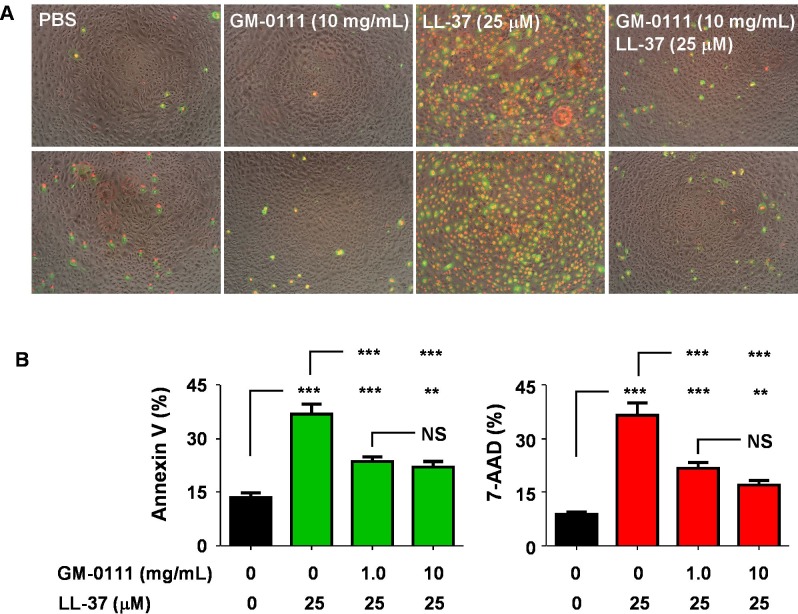# Correction: Prevention of Anti-microbial Peptide LL-37-Induced Apoptosis and ATP Release in the Urinary Bladder by a Modified Glycosaminoglycan

**DOI:** 10.1371/annotation/0b7d0567-2fbd-4d32-84f1-9a0a9c290e70

**Published:** 2014-01-10

**Authors:** Won Yong Lee, Justin R. Savage, Jianxing Zhang, Wanjian Jia, Siam Oottamasathien, Glenn D. Prestwich

Errors were introduced during the preparation of this article for publication. In the second paragraph of the Data Analysis section of the Materials and Methods section, there is an error in the fourth sentence. The correct fourth sentence of this paragraph is: "The data points fell under below detection limit (BDL) were substituted with calculated numbers: detection limit divided by the square root of 2, an imputation method used by Hornung and Reed [28]."

Additionally, in Figure 9 the label 'B' has been omitted. The correct version of Figure 9 can be viewed here: 

**Figure:**