# Supplementary material for: Prevention of Anti-microbial Peptide LL-37-Induced Apoptosis and ATP Release in the Urinary Bladder by a Modified Glycosaminoglycan
Source: PLoS One. 2013 Oct 30;8(10):e77854. doi: 10.1371/journal.pone.0077854 (PMC3813730; doi:10.1371/journal.pone.0077854)
Supplement: Table S1 — Chemical structures of glycosaminoglycan analogs. (DOCX) [file pone.0077854.s001.docx]

| **Compounds** | **Structures (repeating units)** | **Functional groups** | **Molecular Weights (Da)** | **References** |
| --- | --- | --- | --- | --- |
| **GM-0111** |  | R = SO_3_^-^ or H | 5000 | 1 |
| **Heparin** |  | R = H or SO_3_^-^,  R' = CCH_3_ or SO_3_^-^ | 17000-19000 | 2 |
| **Chondroitin sulfate**  (chondroitin-4-sulfate:chondroitin-6-sulfate = 1:0.33) |  | Chondroitin-4-sulfate:  R_1_ and = H  R_2_ = SO_3_^-^ | 15000-20000 | 2, 3 |
|  |  | Chondroitin-6-sulfate:  R_1_ = SO_3_^-^  R_2_ and R_3_ = H |  |  |
| **Pentosan polysulfate** |  | R = SO_3_^-^ | 5700 | 4 |

**References**

1. Prestwich GD, Zhang J, Kennedy TP, Rao NV (2012) Alkylated semi synthetic glycosaminoglycosan ethers, and methods for making and using thereof. United States Patent, US8329673 B2.

2. Manufacturer's Product Information (1996) Heparin Sodium (Sigma H3393 and H9399) and Chondroitin sulfate sodium salt from shark cartilage (C4384).

3. Igarashi N, Takeguchi A, Sakai S, Akiyama H, Higashi K, et al. (2013) Effect of Molecular Sizes of Chondroitin Sulfate on Interaction with L-Selectin. Int J Carbohydr Chem 2013, Article ID 856142, doi:10.1155/2013/856142.

4. Kumagai K, Shirabe S, Miyata N, Murata M, Yamauchi A, et al. (2010) Sodium pentosan polysulfate resulted in cartilage improvement in knee osteoarthritis - An open clinical trial-. BMC Clin Pharmacol 10: 7. doi:10.1186/1472-6904-10-7.
